# Moral uncertainty: A case study of Covid-19

**DOI:** 10.1016/j.pec.2021.07.022

**Published:** 2021-11

**Authors:** Trisha Greenhalgh

**Affiliations:** Department of Primary Care Health Sciences, University of Oxford, Radcliffe Primary Care Building, Woodstock Rd, Oxford OX2 6TD, UK

**Keywords:** Moral uncertainty, Covid-19, Utilitarian ethics, Deontological ethics, Practical ethics, Feminist philosophy

## Abstract

**Background:**

Most writing about uncertainty in healthcare has addressed empirical uncertainty – that is, resulting from insufficient or conflicting facts.

**Objective:**

To consider moral uncertainty by exploring how different theories apply to a single clinical case.

**Method:**

In this philosophical reflection, I briefly acknowledge empirical uncertainty before introducing and exploring the topic of moral uncertainty – defined as the question of what to do when we do not know what (morally) to do—using a case study of my own mother’s deterioration and death from Covid-19.

**Results:**

I identify and apply a number of philosophical theories relevant to managing moral uncertainty, including utilitarianism, deontology, practical rationality and feminist philosophy.

**Conclusion:**

Different moral theories lead to different conclusions about the best course of action in situations of moral uncertainty.

**Practice implications:**

Detailed analysis and close reading of a single case can provide insights into how to act in morally complex situations, but learning is in the form of enriched understanding, not formulaic rules.

## Highlights

• Analyses a single clinical case of death from Covid-19.
• Considers moral uncertainty from multiple philosophical perspectives.
• Finds that different theories may lead to different decisions and actions.

## 1 Introduction

This is not the article I planned to write.

My original plan was to address scientific uncertainty in the context of the pandemic. A new respiratory disease, whose first known case was documented in late November 2019, was quickly traced to a novel coronavirus. But at the start of 2020, almost nothing else was known about it. During that year, scientists mobilised and collaborated like never before to build the evidence base for monitoring, diagnosing and treating Covid-19. By the end of January 2020, before the disease had even been officially named, the virus’s genetic code had been sequenced [1]. Days later, the receptor to which the virus binds was identified as ACE-2 [2]. Diagnostic tests, randomised controlled trials of treatments, and mathematical models of how the virus spreads quickly followed [3]. As 2020 drew to a close, at least 15 therapies had been tested in high-quality randomised controlled trials [4] and seven different vaccines had been approved around the world [5]. Some topics, especially those relating to public behaviour (such as whether and when face masks should be worn [6]) remained controversial. But overall, the story of how scientific uncertainty around Covid-19 was reduced at pace and scale would have been well worth telling.

I had also considered writing about clinical uncertainty. Early guidance on acute Covid-19 depicted it as a respiratory illness characterised by cough, fever and shortness of breath [7]. But for patients suspected of Covid-19, this supposedly classical triad of symptoms is neither sensitive (i.e. absence of this combination does not rule it out) nor specific (their presence does not rule it in) [8]. Covid-19 is now known to present in myriad different ways and may be indistinguishable in its early stages from other acute illnesses [9], [10]. Paradoxically, patients with acute Covid-19 whose blood oxygen levels are dangerously low may appear well and not experience a sensation of breathlessness, increasing clinical uncertainty especially when assessing patients remotely [11]. The story of how my team used largely qualitative methods to tap into the emerging intuitive knowledge of front-line clinicians to develop an early warning score for remote assessment of patients with suspected Covid-19 [12] would also have fitted the topic of this theme issue.

But as I will explain, events overtook me.

## 2 Moral uncertainty

I wish to focus for the remainder of this article on moral uncertainty, which Sepielli defines as the question of “what to do when we don’t know what [morally] to do” (page 5) [13]. Sepielli distinguishes *non-normative uncertainty* (uncertainty about verifiable facts), such as whether a patient with Covid-19 pneumonia is likely to benefit from being put on a ventilator, from *normative uncertainty* (uncertainty about the reasons those facts give us), such as whether putting that person rather than some other person on a ventilator during a shortage is morally right.

Dunham et al. highlight the difference between clinical ethics, driven by a deontological principle of duty of care to one’s patient, and public health ethics, driven by a more utilitarian ethic of achieving the greatest good for the whole population [14]. The sheer numbers of patients seeking hospital admission and life support at the height of the pandemic, they argue, has required clinicians to make utilitarian judgements such as whether to discharge sick patients prematurely to make room for even sicker ones; or how to ration ventilators among patients in respiratory failure.

Much has been written on the impacts on healthcare workers of being compelled to take actions in Covid-19 patients that breach their own moral codes [15], [16]. Borges and Litz distinguish between moral dilemmas (being forced to make trade-offs between one set of moral commitments, such as caring for patients, and a competing set of commitments, such as protecting one’s family), moral distress (a concept developed largely in the medical field, focused on the suffering which results from an individual not being able to do what they feel is right in a particular situation) and moral injury (a related concept initially developed in the context of combat, defined as *“the lasting psychological, biological, spiritual, behavioural, and social impact of perpetrating, failing to prevent, or bearing witness to acts that transgress deeply held moral beliefs and expectations”* page 96, citing Litz et al.) [17].

Similar moral traumas may also affect relatives of patients with Covid-19. Guidance from the Scottish Academy, for example, states:

*“When patients are judged to be dying within hours or days, the presence of family at their side for short visits, or longer stays, is vital to palliative and end of life care and a timeless part of the human experience of life and death. It provides comfort not only to the dying patient, but also to those present, and the inability to be present is a source of anxiety, distress and moral injury that may be long-lasting.”* (page 1) [18].

This guidance acknowledges a profound moral dilemma for relatives: because of the contagiousness of SARS-CoV-2, observing one of the simplest and most fundamental of human rituals – gathering at the bedside of a sick loved one – has both individual and societal knock-ons. In such circumstances, says the guidance, rules cannot be rigidly applied; contextual judgements need to be made [18]. The question then arises: *how* should those judgements be made?

## 3 A case study

My interest in moral uncertainty was triggered by a personal experience. My elderly mother was briefly admitted to hospital following a fall at a time when the incidence of Covid-19 locally was high. Five days later, she developed a fever and was soon readmitted with Covid-19 herself. On the day of her admission, my mother said she did not wish to be placed on a ventilator or admitted to the intensive care unit.

The hospital was overwhelmed with Covid-19 cases. More than 50% of patients were testing positive, most of them with a mutant, highly contagious strain of the virus [19]. Many staff were off sick or isolating. Nurses were working far beyond their paid hours and taking few or no breaks. At the time (late December 2020), very few people had been vaccinated.

After several days, we learnt that my mother had been found to have a fractured leg as a result of her earlier fall. We knew that she was alone, confused, crying in pain, and might die in the next few days, either from Covid-19 (given her risk factors, a 50% chance) or as a result of planned orthopaedic surgery (a 20% chance). With each passing day, we gleaned from the fragmented phone reports that my mother was becoming more distressed, more confused, and closer to death. She had named me as her next of kin.

The hospital had a no-visitors policy except for end-of-life compassionate visits. Because of lockdown and shielding policies in place for much of 2020, we had not seen her for nearly a year. My dilemma – a common one [20] – was whether to accept the invitation to visit my dying mother.

On the one hand, I desperately wanted to see her one last time, provide a measure of physical contact to ease her suffering, and say what were likely to be some final words. On the other hand, the chance of me contracting Covid-19 in a hospital where the virus was rife was high. I am a cancer survivor and over 60, and I live with a husband who also has risk factors for poor outcome. We were in a care bubble with other vulnerable relatives. Even if I self-isolated within our home for two weeks, I could not exclude the possibility that I might pass the virus to others. What if my well-intentioned visit led indirectly to the death of someone who would otherwise have remained well?

I was in no scientific doubt that the virus was airborne [21] and highly transmissible [19], and that, therefore, my chances of contracting it in the hospital were extremely high even if I wore a well-fitting mask [6]. But knowing these facts did not resolve the normative uncertainty. As Kant argued in *A Critique of Pure Reason*, moral questions of the form “What should I do?” can never be answered purely by recourse to empirical questions of the form “What can I know?” [22].

## 4 Moral uncertainty - a utilitarian perspective

In his book, *Moral Uncertainty and Its Consequences* [23], philosopher Ted Lockhart argues that we should perform those actions we are maximally confident are morally permissible, with three caveats. Firstly, if – and to the extent that – there are different degrees of moral rightness, we should seek to *maximise the likely moral rightness* of our actions (rather than maximising the probability that we are doing the right thing) – for example, acting to prevent an unlikely but possible disaster (e.g. a death) has greater moral worth than acting in a way that is almost certainly moral but of limited consequence (e.g. giving someone a thank-you gift). Secondly, in situations where we cannot work out which action maximises likely moral rightness (for example, because there is empirical uncertainty), we should choose the action which *most probably* maximises expected rightness. Thirdly, we should maximise the likely moral rightness of *courses of action*, rather than of individual actions (though in my own example, the act of visiting is a one-off).

Applying Lockhart’s principles to my own moral uncertainty, I must first ask: “Is visiting my mother morally permissible?” and “Is *not* visiting my mother morally permissible?”. Neither question has a clear-cut answer. It is of course morally permissible to visit a dying relative – but that permissibility is not absolute (for example, if the act of visiting places others at risk of harm). Not visiting a dying relative, especially one who is in pain and distress and who has previously selected me as the person to whom, above all others, she has declared a kinship bond would normally be morally dubious if not actually impermissible. But my mother is a deeply religious woman who has already chosen to limit her own treatment options so as to free up resources for others. I know she would not want me to risk innocent lives to be with her.

Considering Lockhart’s caveats, to maximise the likely moral rightness of my actions I would need to estimate the extent to which my visiting would reduce my mother’s distress (impossible to tell, since I have insufficient information about her mental state – she may not even be aware of my presence, though perhaps she will recognise me and take comfort), and also the extent to which my visit would lead to me becoming infected (likely high) and then place someone else at risk of Covid-19 and its consequences (likely low if I self-isolate strictly – but not zero). My own (unlikely but possible) death from the disease would have substantial impact on my family. The action which most probably maximises expected rightness, given the uncertainties involved and the seriousness of certain outcomes, seems to favour not visiting.

## 5 Moral uncertainty – a deontological perspective

Lockhart’s principles for resolving moral uncertainty were of limited use to me in my own dilemma because I found them too abstract, too technocratic and too utilitarian. Perhaps this is partly because I trained as a clinician where I was taught to reason deontologically.

Bykvist challenges the utilitarians’ assumption that the rightness or wrongness of an action can be assessed purely in relation to its predicted consequences. Some actions, she suggests (and visiting a dying parent becomes a case in point), may be *intrinsically* right – as others are intrinsically wrong. Virani et al. argue that visitor restrictions to patients with Covid-19 are based uncritically on utilitarian values which sacrifice the wellbeing of patient, family and staff for the (hoped-for) wider good of a more distant community [24]. It is for precisely this reason that such policies are unpopular with, and may be experienced as traumatic by, the parties most proximally affected by them (one article by patient advocates describes visitor restrictions and similar policies as *“riding roughshod over human rights”* [25]).

In a chapter exploring the implications of Kant’s writings on duty, Barber ([26] page 112) describes two fictitious rich philanthropists: the first feels no sympathy with the poor but recognises her duty towards them and hence gives them money, while the second finds an inner pleasure in giving to the poor, and does it for that reason. According to Barber, Kant (a deontologist) would classify only the first of these acts of giving as having moral worth (in his terminology, it shows good will *arising from* duty), while the second is *in accordance with* duty but undertaken for some other motive [26]. Incidentally, a virtue ethicist might disagree, arguing that the second act has greater moral worth because its perpetrator possesses the virtue of kindness; a utilitarian would probably suggest that both acts have equal moral value if the same amount of money changes hands.

If I choose to draw on deontological theory to resolve my moral uncertainty about whether to visit my mother, I must examine whether I am motivated primarily by a commitment, born of filial duty, to relieve her suffering (even if it subsequently increases my own) or by a self-interested desire to achieve some kind of closure for, and prevent moral injury to, myself. If the former, the deontological case begins to stack up in favour of me visiting.

However, also relevant to deontological reasoning is Kant’s categorical imperative: *“I ought never to act except in such a way that I can also will that my maxim should become a universal law”* (Kant I, 1785, cited in Barber [26], page 100). At an individual level the risks of visiting are small and seemingly compensated by the human benefits, but SARS-CoV-2 (the virus that causes Covid-19) does not spread uniformly. Many infected people do not infect anyone else while a small proportion infect many – a phenomenon known as *overdispersion* (κ) of the reproduction number (R) [27]. Because of a very high overdispersion factor, 10% of infectious people, so called super-spreaders, are likely responsible for about 80% of secondary transmissions [28]. Whilst I personally am unlikely to spread the virus (because I understand the need to wear a close-fitting mask and strictly self-isolate for at least 10 days after visiting, and because I live in a large enough house to be able to do so), others following the same course of action may not be willing or able to take the same precautions. Perhaps, then, I have a duty to set an example and stay away.

## 6 Moral uncertainty – a practical rationality perspective

Practical rationality considers moral judgements to be rooted in everyday, situated, social practices. Such judgements are seen not as generated by the objective and dispassionate application of a formal knowledge base but as a practical achievement, constructed by individual actors through moment-by-moment dialogical reasoning about “complex particulars” [29]. Practical rationality embraces not only empirical facts but also situated subjectivity and narrative reasoning [30], [31], [32]. Indeed, suggests Kathryn Montgomery Hunter, it is through the construction and interpretation of stories of human experience that people come to *“make sense of their circumstances and work out […] what is, on the whole, the better thing to do”* [32] (page 308).

Sapielli has explored different approaches for weighing the relative merits of such things as obligation, permission, strength of reasons, subjective versus objective rightness and the incomparability of values in specific situations of moral uncertainty [33]. But nowhere in his lengthy thesis does he mention emotions. Feminist philosophers, in contrast, have argued that emotions are a crucial component of practical rationality.

Martha Nussbaum, for example, begins her seminal book *Upheavals of Thought: The Intelligence of Emotions* with a description of how she was called to the hospital bed of her mother who was unexpectedly dying from a complication of surgery. She booked the next flight but by the time she arrived, her mother had just passed away. Nussbaum describes a sweep of conflicting emotions in the hours before and the weeks after her mother’s death – compassion, fear, grief, anger, guilt and more. She invites the reader to consider what might be called the defining characteristics of emotions:

*“…their urgency and heat; their tendency to take over the personality and move it to action with overwhelming force; their connection with important attachments, in terms of which a person defines her life; the person’s sense of passivity before them; their apparently adversarial relation to ‘rationality’ in the sense of cool calculation and cost-benefit analysis; their close connections with one another, as hope alternates uneasily with fear, as grief, looking about for a cause, expresses itself as anger, as all of these can be the vehicles of an underlying love.”* (page 22) [30].

Above all, says Nussbuam, emotions *do moral work*: they embody judgements about value. We feel emotional about something we care about. Emotions, so often dismissed as irrational, nonsensical and to be controlled and suppressed, are actually evaluative in nature and should be engaged with. We feel an emotion (or not) towards someone or something; that emotion has a nature (grief, guilt, hatred etc), a direction (positive or negative) and a valency (strong or weak). Far from needing to suppress our emotions in order to properly appraise a situation, says Nussbaum, we appraise the situation *with and through* our emotions.

Bringing emotion into the theoretical frame means that subjectivity, gut feelings and kinship and friendship ties can be embraced and theorised rather than rejected as *“murky contaminants to reason”* [34]. A reviewer of Lockhart’s book, Brian Weatherson, proposes a thought experiment – coincidentally also relating to hospital visiting [35]. In a pre-Covid era, an imaginary character, Jane, arrives at a hospital to visit her sick friend. But, following Lockhart’s principles and caveats, she decides to identify the person in the hospital who is most in need of a visit, and visit that person instead of her friend. In a similar situation, few of us would actually divert from visiting our friend to doing the (utilitarian) ‘right’ thing. As Weatherson comments: *“It is no discredit to Jane that she lacks this general desire, and in some cases it may be a virtue”*, since in his view (citing the philosopher Michael Smith), putting aside a valued friend purely to observe an abstract moral principle would count as a moral fetish [35]. From the perspective of feminist philosophy, moral fetishes which over-ride emotions and sideline personal commitments may be vices, not virtues.

The emotions I felt when I heard my mother was dying were similar to those experienced by Nussbaum in a comparable life event. Anger towards everyone who had ignored masking and social distancing guidance, resulting in the surge of cases from which my mother had become infected. Guilt that I had so rarely found time in my busy schedule to visit her before the pandemic cut us off. Compassion for the depth of her suffering. Fury at a nurse’s decision that a doctor could not be disturbed at night even though she was crying out in pain. Anguish that I may not be able to see and touch her one last time.

These emotions, like Nussbaum’s, were powerful – but not all were relevant to resolving my moral uncertainty. Other people’s past flouting of infection control measures was irrelevant to my current dilemma, however angry I was about them. Conversations about pain control were needed – but they could be held by telephone. Other emotions, however, were key to my moral dilemma. Through an emotionally-engaged feminist philosophy lens, the intensity of my guilt over my failure to make time for my mother in the past, the depth of my compassion for her current suffering, and my overpowering desire to make skin-to-skin human contact, seemed to increase the moral worth of any remaining encounters.

Initially, I used this feminist analysis to reason that it would be morally right for me to visit my mother as she lay deteriorating in hospital. But as my husband drove me on the long journey to pay that final visit, I felt a new wave of powerful emotions – this time towards the man who had walked in step with me for 34 years and was committed to supporting whichever choice I made. If I entered the hospital to visit one loved one, I would be consciously and avoidably placing another loved one at risk. As we drove on in sober silence, I felt shame towards myself and a growing protective instinct towards my partner. When we arrived at the hospital, I could not bring myself to go in. Instead of holding my dying mother’s hand, I grasped my husband’s.

## 7 Adjudicating between theories: the need for meta-philosophy

As this case illustrates, moral uncertainty plays out differently depending on which principles and theories one selects. Indeed, a key aspect of normative uncertainty is the theoretical question of *which* moral theories should be applied in which circumstances [36].

Bykvist has explained why rational, rule-driven approaches to resolving moral uncertainty are often unhelpful:

*“We can be uncertain about how to weigh reasons of autonomy against reasons of benevolence,”* she says (page 1), *“not because we lack an understanding of what these reasons are (what autonomy and benevolence mean, whose autonomy and well-being are at stake, and how much autonomy and well-being are at stake, and so on) but because we are not sure which of the reasons is more morally important*.” (page x) [36].

Both Sepielli and Bykvist propose some meta-philosophical approaches to adjudicating between theories in situations of moral uncertainty, which are complex and highly abstract [33], [36]. For the purposes of this article, perhaps the most important point is the warning that we should be reflexive about the temptation to cherry-pick our moral theories to suit the decision we favour [33]. Just because I can find a moral theory which supports my desire to visit my mother – for example, an elementary reading of virtue ethics would justify me showing ‘courage’ in doing so [37], and a similarly elementary reading of deontological ethics might conclude that as a daughter I have duties to my mother which include being present at her deathbed [38] – does not mean that such an act is necessarily morally right, since these readings are naïve and there are numerous philosophical counter-arguments.

## 8 Conclusion

In this article, I have used a single case study to illustrate various moral principles and theories on moral uncertainty, which has received limited academic attention to date. Most literature on the topic is written from a utilitarian perspective and presented, more or less, as a specific application of rational decision science in which a morally conscious person cares seeks to maximise doing right and minimise doing wrong. Just as clinicians who are trained deontologically struggle with utilitarian principles when required to make decisions under conditions of scarcity, relatives of patients with Covid-19 struggle to weigh their filial duties and kinship ties to the sick or dying loved one against their wider commitments to the community.

Alasdair MacIntyre uses the term ‘the divided self’ to refer to how people in institutional roles typically seek to be coldly rational and consciously override their own emotions when making moral judgements, resulting in a potential threat to moral agency [39]. This phrase captures the hollowness I perceived in academic commentaries when I first began to explore my own moral uncertainty. How could Kant assign greater moral worth to the actions of a philanthropist who did not *care* about the poor to whom she gave money than to the actions of someone who did? How could Lockhart depict a visit to a sick friend as worth morally less than a visit to a stranger?

Writers on practical rationality, and particularly the feminist philosophy of Martha Nussbaum, allowed me to transcend this hollowness and use my emotional response to my mother’s predicament – in all its subjectivity and confusion – to inform my analysis of what, morally, I should do. Whilst I initially concluded that it was right for me to visit, I was eventually guided to the opposite conclusion not by a dispassionate appraisal of empirical facts or formal value hierarchies but by a further wave of emotional responses, this time directed towards my husband. This decision aligns with Lockhart’s first caveat about maximising moral rightness (the chance that someone would die as a consequence of my actions was low, but if that did happen it would be very, very wrong).

While exploring this issue, I learnt that resolving moral uncertainty by selecting “my favourite moral theory” is questionable from a meta-philosophical perspective.

## 9 Epilogue

When we arrived at the hospital, my husband and I sat in the car park for three hours. Our adult son, a junior doctor who had been vaccinated against Covid-19, went in and sat at my mother’s bedside during her final hours. He linked us and many other relatives by smartphone video to say our last goodbyes. My mother (who had been given morphine) did not respond, but looked peaceful.

She died, aged 94, that same evening.

*This article is dedicated to the memory of Mrs Mary Greenhalgh 30.8.26–27.12.20 and to the staff at the William Harvey Hospital, Ashford, UK, who cared for her in her final illness.*

## Funding statement

TG’s research was funded by the 10.13039/100004440Wellcome Trust (WT104830MA).

## CRediT authorship contribution statement

Professor Greenhalgh is the sole author.

## Conflict of interest

The author declares no conflict of interest.
